# VarI-SIG 2015: methods for personalized medicine – the role of variant interpretation in research and diagnostics

**DOI:** 10.1186/s12864-016-2721-3

**Published:** 2016-06-23

**Authors:** Yana Bromberg, Emidio Capriotti, Hannah Carter

**Affiliations:** Department of Biochemistry and Microbiology, Rutgers University, Lipman Hall 218, 08901 New Brunswick, NJ USA; Department of Genetics, Rutgers University, Lipman Hall 218, 08901 New Brunswick, NJ USA; Institute for Mathematical Modeling of Biological Systems, Department of Biology, Heinrich Heine University Düsseldorf, Universitaetsstr. 1, 40225 Düsseldorf, Germany; Division of Medical Genetics, Department of Medicine, University of California, San Diego, 9500 Gilman Dr., 92093 La Jolla, CA USA

## Introduction

The growing availability of high-throughput sequencing continues to increase the number of identified genetic variants [1, 2]. For example, the size of the dbSNP database [3] has grown exponentially over the past years to ~150 million human single nucleotide polymorphisms and short genomic variants. Unfortunately, the characterization, annotation, and interpretation of these variants are still lagging. In particular, their implication in disease is one of the major challenges in personalized medicine [4–8].

The 5th edition of the **Var**iant **I**nterpretation **S**pecial **I**nterest **G**roup (VarI-SIG, formerly SNP-SIG) meeting [9–12] was held on July 11th, 2015 at the joint ISMB/ECCB meeting in Dublin, Ireland. The central VarI-SIG themes were “Annotation and prediction of structural/functional impacts of coding variants” and “Genetic variants as effectors of change: disease and evolution”. Our meeting is organized as a venue for the development of a research network of scientists, necessary for facilitating the exchange of ideas and establishing new collaborations. This year’s meeting attracted over 60 participants, with eight research talks and five presentations from the leading scientists in the field.

## Manuscript submission and review

For this year’s VarI-SIG special issue we received eight manuscript submissions. All manuscripts were evaluated by at least two reviewers, selecting from a panel of three editors and 17 other experts in the field (see *Acknowledgements*). After 2 rounds of review seven manuscripts were accepted for publication. These address a description of a phenotype-dependent variant/gene prioritization method [13], annotation of variants specifically in protein kinases [14] and, generically, in regulatory regions [15], analysis of genetic variants associated with Alzheimer’s Disease [16], identification of the role of protein stability OMIM disease-related variants [17], and the study of mutation profiles in cancer genomes [18, 19].

[The complete program of VarI-SIG meeting 2015 with presentation and poster abstracts is available at http://varisig.biofold.org/2015/docs/vari-sig-2015-programme.pdf]

## Further developments

We are working to organize the next VarI-SIG meeting (ISMB 2016; Orlando, Florida; July 9th, 2016). Further information about this coming meeting is available on our website (http://varisig.biofold.org).

Last year, in collaboration with the ISMB organizers, we introduced VarI-COSI (Variant Interpretation Community of Special Interest). VarI-COSI is a community aimed at sharing relevant information, discussing ideas, and providing training and support networks in the field of genomic interpretation. The web portal for VarI-COSI is under development and is accessible via ISCB Connect (http://connect.iscb.org/home). We welcome input and participation from the variation interpretation community.
